# A Comparative Retrospective Study on Surgical Versus Conservative Treatment of Simple Fifth Metacarpal Neck Fractures

**DOI:** 10.7759/cureus.73439

**Published:** 2024-11-11

**Authors:** João Lixa, Luisa Vital, Paula Vieira, Ana Esteves, Miguel Silva, André Pinho, Vítor Vidinha

**Affiliations:** 1 Orthopaedics and Traumatology, São João University Hospital Center, Porto, PRT; 2 Orthopaedics and Traumatology, Centro Hospitalar do Tamega e Sousa (CHTS), Porto, PRT

**Keywords:** clinical outcomes, comparative study, dorsal angulation, fifth metacarpal neck fracture, retrospective study

## Abstract

Introduction

The fifth metacarpal neck fracture is a common injury, and the treatment of such injuries is still a matter of debate. It typically presents in young adults of working age, and it represents a significant burden for both the health services and the patient. The purpose of this study was to compare the results of operative and conservative treatment of this fracture.

Materials and methods

A retrospective study of 60 patients with a fifth metacarpal neck fracture was conducted. The sample was divided into two groups: patients treated operatively and non-operatively. The mean angulation in the different groups was compared using the t-test, and the variance achieved by manipulation or surgery was compared using the paired t-test. The QuickDash questionnaire was applied.

Results

The mean initial dorsal apex angulation was 53° in the surgical group and 45° in the conservative group. The mean angulation at discharge was 28° in the surgical group and 38° in the conservative group. The QuickDASH questionnaire results were similar between groups, with the surgically treated patients reporting more pain and less aesthetic satisfaction.

Conclusion

This study corroborates the growing evidence favoring expanding conservative treatment to most of these fractures.

## Introduction

The fifth metacarpal neck fracture is a very common injury, representing 20% of all hand fractures [1-3], most commonly as the result of a direct trauma with a clenched fist, hence the term Boxer's fracture [3,4]. Axial force applied to the metacarpal results in apex dorsal angulation by the pull of the interosseous muscles [5]. Comminution of the volar aspect of the neck is often present, as it represents one of the weakest points in the metacarpal [5].

It typically presents in young male adults of working age [6,7] and it represents a significant burden for both the health services and for the patient, resulting in days off work, consumption of resources, and potential poor hand function [8,9].

Initial cadaveric studies [1,10] suggested that dorsal apex angulation caused shortening of the metacarpus and of the flexor digitorum profundus tendon, resulting in limited joint motion. The accepted angulation for conservative treatment was then fixed at 30° degrees [1,11]. Newer clinical studies, basing their findings on muscular compensatory mechanisms in vivo, suggested increasingly higher dorsal angulations up to 60-70° as acceptable for conservative treatment [9,12,13].

Despite the treatment of these injuries still being a matter of debate, some recent systematic reviews and meta-analyses also favor conservative treatment for most patients [2,9,13,14].

Open fractures, pseudoclawing, or rotational deformity remain formal indications for operative management [5,14].

The purpose of this study is to compare the results of operative and conservative treatment of a closed fifth metacarpal neck fracture without rotational deformity.

## Materials and methods

**Figure 1:**
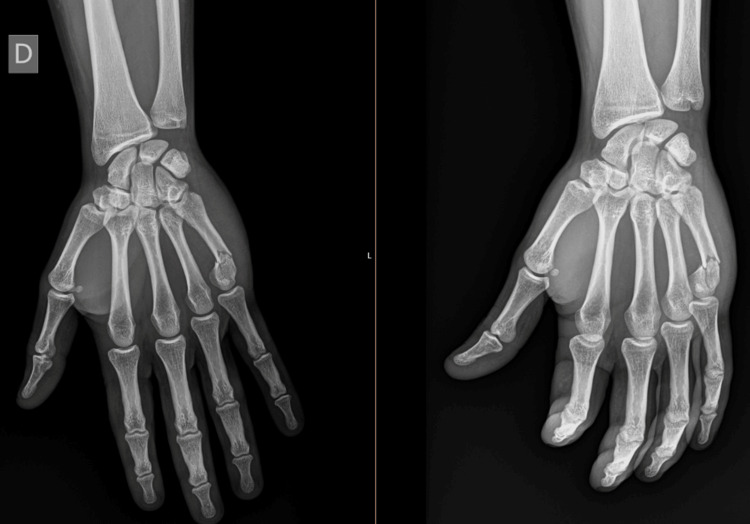
Fifth metacarpal neck fracture

We conducted a retrospective study from July 2021 to September 2021 of patients with a fifth metacarpal neck fracture treated in Centro Hospitalar Universitário de São João, Porto, Portugal (Figure 1). We reviewed all cases with an isolated fifth metacarpal neck fracture, classified as 77.5.3A according to the OTA classification [15], with at least a year of follow-up since trauma admission between 2018 and 2019. We included 60 consecutive patients, 30 treated operatively and 30 non-operatively. Open fractures, multi-fragmentary fractures, pseudoclawing, or fractures with rotational deformity were excluded. 

**Figure 2:**
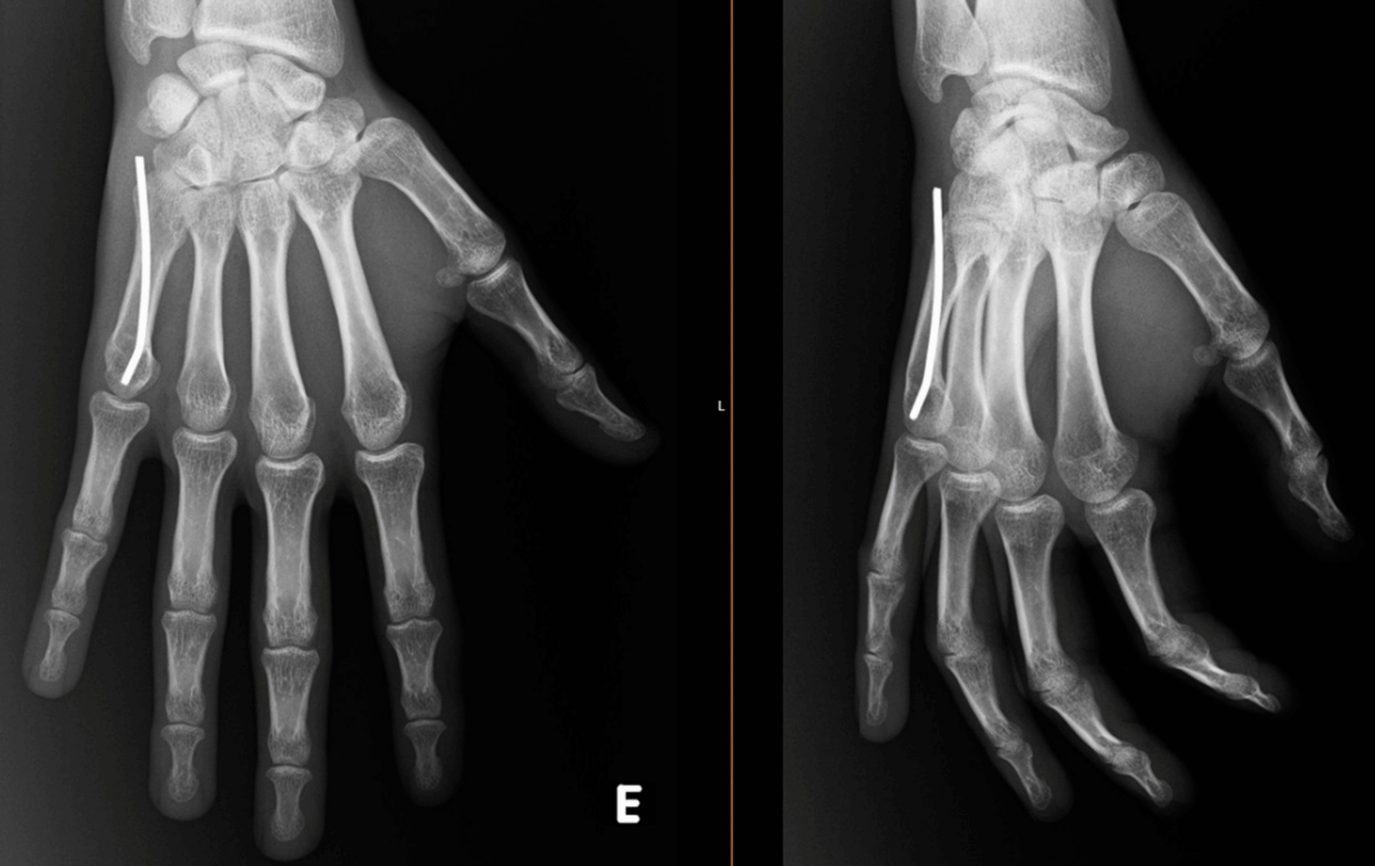
Percutaneous antegrade intramedullary pinning with Kirschner wire

In the non-operative group, closed reduction was performed followed by ulnar casting for a period of three weeks. In the operative group, the closed reduction under anesthesia was followed by percutaneous antegrade intramedullary pinning stabilization with one or two Kirschner wires (Figure 2). This group was also immobilized with an ulnar casting for a period of two weeks. The rehabilitation protocol and hand exercises prescribed were similar in both groups. Kirschner wire extraction was performed in all patients at 6 to 12 weeks postoperatively, depending on callus formation.

Demographic information was collected, and radiographic parameters were analyzed. We determined the dorsal apex angulation in the lateral view at several moments: at admission, after closed manipulation and casting in the cases of conservatively treated patients, at postoperative in the surgically treated patients, and at discharge in both groups of patients. The radiographic measurements were performed in a standardized side view radiograph performed as a protocol in fifth finger trauma in our institution by two of the authors (JL and PV) and presented as a mean between the two measurements. Then, with at least a year since the trauma, we applied the QuickDash questionnaire [16], the numerical pain scale in daily activities [17], and a two-item questionnaire designed for this study to assess overall functional and aesthetic satisfaction (Table 1).

**Table 1:** Functional and aesthetic satisfaction questionnaire

	Yes	No
“Are you overall satisfied with the function of your affected hand?”		
"Are you overall satisfied with the aesthetics of your affected hand?”		

The sample was divided into two groups: patients treated operatively and non-operatively.

Chi-square analysis was used for the determination of differences between nominal variables and parametric tests for continuous variables with normal distribution. Paired t-test analysis was used for paired continuous variables with normal distribution, and non-parametric tests were used for variables with non-normal distribution.

The local IRB approved this study (number 363-21), and the patients gave their consent to be included in the study as the questionnaires were applied.

Statistical significance was considered for p values < 0.05. Statistical analysis was performed using IBM Corp. Released 2021. IBM SPSS Statistics for Windows, Version 28.0. Armonk, NY: IBM Corp.

## Results

The mean age of our study sample was 37 years old, and 87% were male, with no differences found between groups. The mean initial dorsal apex angulation was 53° in the surgical group and 45° in the conservative group (p=0.041) (Table 2). 

**Table 2:** Distribution of mean age, gender, and mean values of initial and final angulation between groups

	Conservative group (n=30)	Surgical group (n=30)	Statistical test value	p-value
Age (years)	39	36	0.85	0.404
Gender (male)	87%	87%	0.99	0.507
Initial angulation	45°	53°	-2.09	0.041
Final angulation	38°	28°	2.86	0.012

The mean angulation at the moment of discharge was 28° in the operative group and 38° in the non-operative group (p=0.012). In the latter group, we found that the mean angulation at admission was statistically higher than at discharge (45° vs. 38°; p<0.001) (Table 3), with conservation of the post-manipulation reduction at discharge (p=0.267) (Table 4). At the follow-up period of one year, there were no cases of nonunion. 

**Table 3:** Comparison of mean values of initial and final angulation inside conservative and surgical groups Delta: Difference in mean values between initial and final angulation in each group

	Initial angulation	Final angulation	Delta	Paired t test value	p-value
Conservative group (n=30)	45°	38°	7°	2.81	p<0.001
Surgical group (n=30)	53°	28°	25°	6.89	p<0.001

**Table 4:** Mean values of post reduction and post operative angulation, for conservative and surgical group respectively, and mean values of final angulation in each group. Delta: difference in mean values between post reduction and post operative angulation and final angulation in each group

	Post reduction/post operative angulation	Final angulation	Delta	Paired t test value	p-value
Conservative group (n=30)	36°	38°	2°	-1.31	0.267
Surgical group (n=30)	27°	28°	1°	-0.14	0.320

Regarding the interviews, the surgical group had a superior mean numerical pain scale score (0.1 vs. 1.5; p=0.02), although in the QuickDash there were no differences found (p=0.630). The overall functional satisfaction was 100% in both groups, and the aesthetic satisfaction was inferior in the surgical group (71% vs. 100%) (Table 5). The main reason reported for aesthetic dissatisfaction was a prominent scar.

**Table 5:** Results of the questionnaire applied.

	Conservative group (n=30)	Surgical group (n=30)	T-test value	p-value
Numerical pain scale	0.1	1.5	-2.44	0.020
QuickDash score	14	16	-0.49	0.630
Functional satisfaction	100%	100%		
Aesthetics satisfaction	100%	71%		

## Discussion

The fifth metacarpal neck fracture remains a common pathology presenting to health services worldwide [7,8]. Similar to what is described in the literature [18], our sample is composed mainly of young male adults of working age, 87% males with a mean age of 37 years.

The best treatment of closed fractures without rotational deformity remains a matter of debate in the orthopedic community [2,3,9]. There are several non-operative treatment strategies, such as ulnar casting or buddy taping with or without closed reduction first. A Cochrane systematic review by Poolman et al. [19] analyzed several methods of non-operative treatment and reported no superiority of any method regarding radiographic and clinical outcomes. Muller et al. [20] found no differences regarding the range of motion, pain, and patient satisfaction between patients treated with either an ulnar gutter cast for 3 weeks followed by mobilization or a pressure bandage for 1 week and immediate mobilization, with angulations smaller than 70°. They found that immediate mobilization presented good results and satisfied patients. Hansen et al. [21] found similar results in patients with less than 60° of angulation and recommended a functional brace because patients became mobile faster and experienced less pain.

In this study, the mean initial angulation was superior in the surgical group (p=0.041), confirming the tendency for proposing surgery with higher angulation values. Still, the initial mean value of this group (53°) was slightly lower than the now commonly accepted reference of 60° [9,12,13]. This might be explained by differences in the surgeon’s preference regarding these injuries or by the patient's choice of surgical treatment.

Closed reduction of the fracture aims to reduce the dorsal angulation and restore the functional anatomy of the finger without disruption of the tissues and the fracture hematoma. The improvement of angulation achieved in this study with manipulation in the non-operative group was 7°, similar to other studies [22,23]. This amount of improvement questions the benefit of closed manipulation, as the small change in angulation doesn't seem to represent clinical significance [23]. The reduction achieved in the surgical group was 25°, traducing greater power of reduction when the patient is under anesthesia, direct radiographic control, and immediate fixation.

The stability of the fracture is determined by the degree of initial displacement and comminution. In our study, both groups did not register any significant loss of reduction between the post-reduction or postoperative period to the last follow-up, in the conservative and surgical groups, respectively. This suggests that regardless of method, the reduction achieved is usually well maintained, findings similar to those reported by Kaynak et al. and Kuokkanen et al. [22,24].

The outcomes reported by the patients were similar in both groups, except regarding residual pain and aesthetics. QuickDash scores presented no differences (p=0.630) and the question regarding functional satisfaction was 100% in both groups. The correlation between dorsal angulation and clinical outcomes was also studied by other authors [20,25], without any significant findings. Kuokkanen et al. [24] reported good outcomes without disability with 70° of angulation. Lowdon [26] failed to report a relationship between the presence of symptoms and residual angulation in fifth metacarpal neck fractures. Extensor lag also has been shown to improve over time, with 94% of patients achieving contralateral grip strength by 1 year in one study [27]. Two prospective randomized controlled trials [25,28] compared nonoperative and surgical management and reported no differences in outcomes at 12 months. QuickDASH, visual analog scale (VAS), reported range of movement (ROM), and mean grip strength were similar in both groups. In fact, patients in the surgical group were three times more likely to suffer complications like neurological symptoms (chronic pain, paraesthesias), infection, or complex regional pain syndrome. Strub et al. [28] even reported a longer period of work in the group of patients treated with percutaneous antegrade intramedullary pinning with Kirschner wires. In our study, the surgical scar seems to represent greater aesthetic discomfort than the deformity caused by the angulation of the fracture. The residual pain may be caused by the scar, the presence of the foreign material, or the manipulation of the tissues and nervous structures. These findings, in addition to a 100% functional satisfaction rate in both groups, suggest a superiority of the conservative treatment regarding similar functional outcomes with fewer complications.

Boulton et al. [29] described an intramedullary fixation using cannulated headless screws for a more rigid construct. This technique is indicated in displaced and unstable fractures and permits a smaller immobilization period, intending to reduce the social burden of these lesions [30]. Nonetheless, there are studies [30] reporting similar results in terms of range of motion, grip strength, satisfaction, postoperative pain, and Quick DASH scores between intramedullary pinning with Kirschner wires and intramedullary screw fixation. Thus, this technique still requires studies to prove efficacy given the higher cost, risk of chondral damage, and retention of hardware compared to other techniques.

Although good results are achieved by non-operative treatment, not all fractures can be managed without surgery. Pseudoclawing, hyperextension of the metacarpophalangeal joint, and flexion of the proximal interphalangeal joint are the results of a compensatory response to the imbalance between the longer extensors and shorter intrinsic muscles caused by the shortening and the apex dorsal angulation [5]. It is functionally unacceptable and remains an indication for operative treatment. Rotational deformity of the finger, open or severely comminuted fractures, and fractures of several metacarpals constitute other indications for surgery.

The mean initial angulation in the surgical group of 53° suggests that our department has a lower threshold for surgery in these patients. We speculate that this may be the case in other departments: in daily practice, the classic literature is still taken into consideration for decision-making. As discussed previously, the current literature suggests that a 60° or more deformity produces good clinical results without the risk of surgical complications [20,25]. A shift towards conservative treatment with higher angulations will come as no surprise, as more studies like this one are carried out and published.

Although the final mean angulation was inferior in the surgical group, the difference of 10° was statistically (p=0.012) but not clinically significant. The functional satisfaction and the QuickDASH questionnaire were similar, with surgically treated patients reporting more residual pain and less aesthetic satisfaction.

The main limitation of this study is the lack of functional outcome measurement like range of motion or grip strength; only QuickDASH and satisfaction questionnaires were applied. Furthermore, the reproducibility of the radiographic measurements is difficult because of the variance in the radiographic views in which the angulations are measured [13]. This may introduce an error that is expected to be similar in both groups.

## Conclusions

The relation between the angular deformity of the fifth metacarpal neck fracture and functional outcomes is not yet well established. Although the final mean angulation was inferior in the surgical group, the difference of 10° was statistically (p=0.012) but not clinically significant. The functional satisfaction and the QuickDASH questionnaire were similar, with surgically treated patients reporting more residual pain and less aesthetic satisfaction.

This study corroborates the growing evidence favoring expanding conservative treatment to most of these fractures, suggesting a small role of dorsal angulation in the final result of these injuries. Surgical treatment should be reserved for complex fifth metacarpal neck fractures.
